# Hormonal induction of spermiation in a Eurasian bufonid (*Epidalea calamita*)

**DOI:** 10.1186/s12958-019-0537-0

**Published:** 2019-11-11

**Authors:** Lucía Arregui, Sergio Diaz-Diaz, Elia Alonso-López, Andrew J. Kouba

**Affiliations:** 1 Department of Biology, Universidad Autónoma de Madrid, C/Darwin 2, Av. Niceto Alcalá Zamora, 19, 4°2, 28050 Madrid, Spain; 2 Department of Wildlife, Fisheries and Aquaculture, Mississippi State University, 775 Stone Boulevard, Starkville, MS 39762 USA

**Keywords:** Spermatozoa, Human chorionic gonadotropin, Anuran, Toad, Ex situ conservation

## Abstract

**Background:**

Amphibian diversity is declining at an alarming rate due to habitat loss, invasive species, climate change and diseases. Captive assurance colonies have been established for some species at risk; however, many species do not breed well in captivity and the development of assisted reproductive technologies (ART) is critical to help sustain genetic diversity. To date, the majority of the work has been accomplished in species from the American continent and Australia, and there is a need to address similar breeding challenges in Eurasian and African species of amphibians.

**Methods:**

The aim of this study was to develop a hormone protocol for stimulation of spermiation in *Epidalea calamita* as a model for Eurasian bufonids. Hence, the effect on sperm production and quality of three doses of chorionic gonadotropin hormone (5, 10 and 15 IU hCG/g) over time (1 to 24 h) was evaluated. In addition, cold storage (at 5 **°**C) of sperm for 24 and 48 h and three frequencies for hormonal treatment (weekly, biweekly and monthly) were examined.

**Results:**

Hormone concentrations of 10 or 15 IU of hCG induced spermiation in 100% of males and produced sperm of comparable quality, while 5 IU hCG stimulated spermiation in only 40% of males. Total motility peaked between 1 to 4 h post-treatment with 10 IU hCG, whereas treatment with 15 IU hCG peaked between 2 to 6 h. After 24 h of cold storage total motility dropped by 20% and forward motility dropped by 10% for both the 10 and 15 IU treatments. Weekly hormone administration resulted in higher variation between trials in all motility parameters and a lower overall Total Motility and Forward Movement. Furthermore, the effect of exogenous hormone treatment overlapped between the last two trials in the weekly frequency. Sperm concentration was higher in the first trial for all frequencies but showed no differences among other trials.

**Conclusions:**

Overall, these results show that hormone concentration, time after treatment, frequency of hormone treatment and cold storage should be borne in mind when developing a hormone stimulation protocol for Eurasian amphibian species.

## Background

Establishment of in-situ and ex-situ conservation actions are urgent for at risk amphibian species. Amphibians are currently facing an extinction crisis with 40% of known species under some degree of threat. Little progress had been made in relation to the looming scale of the crisis [1] even though alarms and dire warnings were raised close to thirty years ago during the first workshop on Declining Amphibian Populations [2, 3].

The important role of assisted reproductive techniques (ART) for the conservation and genetic management of threatened species has been highlighted in several reviews [4–8]. The implementation of these technologies for threatened amphibian species has been prioritized within recovery programs in order to address the low reproductive output and steady decline of founder lines in captivity [9]. For amphibians, hormone therapy is the first step for the development of ART as it can promote stimulation of natural breeding behaviour (e.g. amplexus) and facilitate the non-lethal collection of gametes for artificial fertilization and/or cryopreservation.

The Bufonidae family of amphibians has a worldwide natural distribution, except for Australia and Antarctica, and is the second most diverse family with more than 535 species; 43% of bufonid species are threatened and 22% are considered rapidly-declining species [10, 11]. Nearly 58% of bufonid species are native to the American continent while the remaining 42% are distributed evenly between Eurasia and Africa [11]. To date, studies developing reproductive biotechnologies in bufonids have mainly focused on species from the American continent such as some species of the genus *Anaxyrus, Incilius and Rhinella* [12–16]. Unfortunately, there is a dearth of knowledge on the reproductive biology and ART for Eurasian bufonids precluding its application for conservation purposes.

Optimal hormone concentrations and timing of their administration for stimulation of spermiation have been shown to differ between species. It has been suggested that related species seem to respond similarly to the same hormones, however, appropriate hormone concentrations must be tested [17, 18]. Sperm have been obtained after hormonal treatment from at least 10 species of bufonids originating from the American continent, these are: *Anaxyrus americanus, A. baxteri, A. boreas, A. fowleri, A. houstonensis, Atelopus zekeri, Incilius valliceps, Rhinella arenarum, R. marina* and *Peltophryne lemur* [13, 16, 19–25] and one from Europe and north of Africa; *Bufo bufo* [11, 26]. Two primary hormones that have shown success in stimulating spermiation in bufonids include Gonadotropin releasing hormone (GnRH) or its synthetic analog called Luteininzing hormone-releasing hormone (LHRH) [16, 19–22, 24–27] and human Chorionic Gonadotropin (hCG) [13, 16, 22, 25]. In direct comparison to each other, a higher concentration of sperm was found in hormonal therapy using hCG for *R. marina* [22] and *A. americanus* [16] and with GnRH in *A. zeteki* [25]. Some studies have analysed the effect of different hormone concentrations over time on sperm quantity and quality [16, 22, 25, 26]. Understanding gamete production and quality over time are important, as synchronization of gamete release from both sexes is necessary to increase fertilization success. For example, when male and female *A. boreas* were treated simultaneously resulting in a low percentage of fertilized eggs, possibly due to asynchrony of sperm and egg release [23]. Another reason to study sperm production over time following hormone administration is that sperm samples with better quality could be selected for artificial fertilization, genetic resource banking, short-term cold storage or studies related to reproductive biology of the species.

Spermatozoa of some anurans can be held at 4–5 °C for short periods and retain viability and motility for days to weeks [15, 28–30]. Having this flexibility of cold storage allows shipping of sperm samples to other institutions to perform artificial fertilization or cryopreservation, rather than moving or capturing the animals, connecting different populations. Therefore, whenever development of ART occurs for a new species it is important to have an understanding of the impact cold-storage has on sperm quality and viability over time. Likewise, understating how frequently you can administer hormones to an animal before if become irresponsible or before having negative effect on sperm quality is valuable to know, especially where hormones are obligatory to support reproduction. Previous research has shown variation among species. Fertilization success declined on the third day of successive hormone injections for the same males in *Rana sylvatica* [31]. In contrast, there was no effect on fertilization success for *Lithobates pipiens* when sperm were obtained after repeated hormonal treatment, although hormone treatments were separated by 4 to 10 days and hormone concentration used was lower [32]. In *R. marina* and *A. fowleri,* induction of spermiation twice a week resulted in less sperm concentration compared with treatment once a week or every other week [33, 34]. Similarly, *Lepidobatrachus laevis* showed a drop in spermatozoa production when treatments were separated by 5 days; yet, no effect was observed when repeated treatments were separated by 23 to 40 days [35].

Prior to development and implementation of ART protocols for threatened Eurasian bufonids, a strategy should be the advancement of knowledge in a related model species such as the Natterjack toad (*Epidalea calamita*). This species is a good model because of its medium size making it easy to work with (e.g. administer hormone injections) and wide distribution across Europe from the Iberian Peninsula to Ukraine and Belarus. The global conservation status of this species is regarded as Least Concern [36]; however, populations are decreasing, mainly in the northern part of its range where it is considered endangered, due to habitat loss and fragmentation [37–40]. The objectives of this study on *E. calamita* were to: (1) test three different concentrations of hCG over time on sperm production; (2) assess short-term cold storage on sperm quality; and (3) evaluate the influence of hormone treatment frequency on quantity and quality of sperm production. Results from this study will elucidate aspects of *E. calamita* reproductive physiology and develop protocols that could be applied to Eurasian bufonids that are threatened with extinction.

## Methods

### Animal maintenance

*Epidalea calamita* males were caught from the wild in Hoyo de Manzanares (Madrid, Spain) during October and November 2011 for experiments 1 and 2 and in September and October 2012 for experiment 3. Collection permits were provided by Comunidad de Madrid (10/420609.9/11 and 10/341608.9/12). Toads were housed in single-sex groups in plastic tubs (50 × 35 × 40 cm). Natural photoperiod (e.g. lights on timers), water and hide boxes were provided. Animals were fed mealworms and adult crickets dusted with calcium powder twice a week. Prior to the start of any experiment, males were weighed for calculating the hormone concentration to be administered and snout-vent length (SVL) was measured using callipers.

### Spermic urine collection and assessment

For induction of spermiation, males were given an intraperitoneal injection of hCG (Sigma, Madrid, Spain) diluted in PBS (Gibco, Madrid, Spain). Urine was collected prior to hCG administration in all the experiments to verify the absence of spermatozoa. Spermic urine was obtained from all males by holding the animals over a petri dish until urination. If spermic urine could not be obtained by handling, a flexible vinyl catheter (outside diameter 1.32 mm) was gently introduced in the cloaca to drain the urine. The volume of urine was measured using a pipette. Following urine collection, toads were returned to their plastic enclosure, which contained 2 cm of water so as to encourage water absorption and urine production. All urine samples were assessed for presence of sperm; if sperm were present, motility and concentration were evaluated at 400x magnification on an Olympus CH2 microscope. One hundred spermatozoa in randomly selected fields were counted to quantify the percentage of (1) sperm with forward movement, (2) sperm with flagellar movement but stationary and (3) non-motile sperm. The total motility was calculated as the addition of sperm with forward movement plus sperm not moving forward but showing flagellar activity. In addition, the quality of motility was evaluated and is a subjective value between 0 and 3 (where 0 = no sperm moving, 1 = < 25% moving vigoursly, 2 = 25–75% moving vigoursly and 3= > 75% showing fast and straight progression). Sperm concentration in each sample was measured using a Neubaeur hemocytometer.

### Experiment 1: Effect of hCG concentration and time post-treatment on sperm production and quality

For induction of spermiation, three treatment groups (*n* = 5 males/group) were established consisting of 5, 10 and 15 IU hCG/g of animal body weight (BW). Volume of diluted hormone varied depending on weight and ranged from 110 to 335 μl. A negative control (*n* = 3 males) was established and toads were treated with the correspondent volume of carrier saline (PBS). Spermic urine was collected from all males prior to hormone administration (time 0) and every hour post-administration (1, 2, 3, 4, 5, 6, 7, 8 and 9 h) with a follow up collection 24 h post-hormone administration. The presence of sperm was noted and quality/quantity assessed as described above.

### Experiment 2: Effect of cold storage on sperm parameters over time

All sperm samples collected in experiment 1 were kept in the refrigerator at 5 **°**C and evaluated 24 and 48 h later. In the case of samples obtained at the 24 h time point, review of cold-stored samples occurred after 24 h of refrigeration. Prior to analysis, stored sperm was mixed, a sub-sample was removed, and sperm parameters were assessed similar to above in experiment one, except concentration was not evaluated.

### Experiment 3: Frequency of hCG treatment on sperm parameters

The effect of hormone administration frequency on sperm parameters was evaluated by establishing three treatment groups (*n* = 7 males/group), where each treatment decreased in frequency of hormone administration. Treatment 1 toads were administered hormone once every 7 days (called weekly), treatment 2 toads were administered hormone once every 14 days (called biweekly) and treatment 3 toads were given hormone once every 28 days (called monthly). Each treatment was administered 5 times in sequence (trials), thus, treatment 1 lasted 5 weeks, treatment 2 lasted 9 weeks and treatment 3 lasted 17 weeks. All males were injected with 10 IU hCG/g BW based on results from experiment 1. Spermic urine was collected at 1, 2, 3 and 4 h post-hormone administration and sperm parameters analysed as described above.

### Statistical analyses

**Fig. 1:**
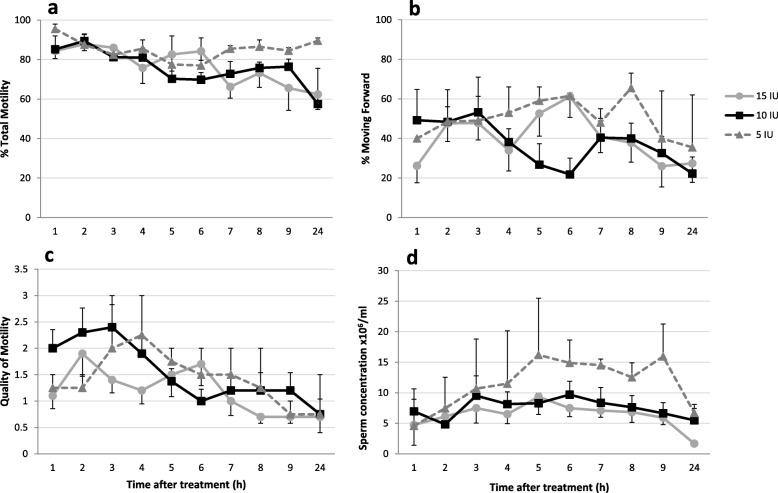
Sperm parameters from male toads over time after treatment with three different concentrations of hCG (5, 10, 15 IU/g BW). **a** Percentage of total motility, **b** percentage of sperm moving forward, **c** quality of motility and **d** sperm concentration. Values are means ± SEM. *N* = 5

Data analysis was performed with SPSS 23 for Windows (SPSS Inc., Chicago, IL, USA). Animal weight and SVL was compared by ANOVA between treatments in experiment 1 and 3 and using a paired T-test between the first and the last trial in experiment 3. In addition, an ANOVA was used to analyse differences on sperm parameters among groups in the first session of experiment 3. Data were analysed using the Generalized Estimating Equations (GEE). Sperm quality (forward movement, total motility and quality of motility) and quantity (concentration) was evaluated with a linear model while the proportion of males presenting sperm in experiment 3 was compared using a binary model. Because we only obtained spermatozoa from two animals treated with 5 IU hCG in experiment 1, these data were not used for analysis, but are represented in Fig. 1. To study the effect of hormone concentrations and time post-hormone treatment, both variables were introduced as factors and time post-hormone administration was treated as a within subject variable. For experiment 3, the data mean for the four time points was calculated and used for analysis. To study the effect of frequency and trial sequence on sperm production both were introduced as factors and trial was treated as a within subject variable. Moreover, a pairwise comparison using Bonferroni correction was performed. Data are expressed as mean ± SEM and a *p* < 0.05 was considered significant.

## Results

### Experiment 1: Effect of hCG concentration and time post-treatment on sperm production and quality

There were no differences (*p* > 0.05) in the weights or SVL between the male toads randomly assigned to one of the three treatment groups (Table 1). None of the 18 males presented sperm in urine prior to hormone treatment and the three control males, treated with PBS only, had no sperm during the entire experiment. Two out of the five males (40%) treated with 5 IU hCG/g produced sperm, whereas 100% of male toads treated with 10 or 15 IU hCG/g produced sperm. All animals that responded to hormone treatment by producing sperm did so beginning at the first time point (1 h). Moreover, all males but one (in 10 IU hCG/g treatment group), had sperm at 24 h post-hormone administration.

**Table 1:** Weights and lengths of toads in each experiment

	Group		Weight (g)	Length (cm)
Experiment 1	5 IU		25.4 ± 3.0	6.75 ± 0.28
	10 IU		27.8 ± 3.0	6.48 ± 0.27*
	15 IU		26.6 ± 2.2	6.43 ± 0.19
Experiment 3	Weekly	Trial 1	28.9 ± 3.1	6.71 ± 0.29
		Trial 5	29.5 ± 2.7	
	Biweekly	Trial 1	28.2 ± 2.8	6.81 ± 0.19
		Trial 5	26.4 ± 2.1	
	Monthly	Trial 1	24.7 ± 2.2	6.57 ± 0.20
		Trial 5	26.1 ± 1.7	

*N* = 6 in experiment 1, *n = 5, n = 7 in experiment 3.

The models showed that hormone concentration was not significant, although time post-hormone treatment and the interaction of concentration and time were significant predictors (*p* < 0.001) for the four sperm parameters analysed (total motility, forward motility, quality of motility and concentration). In general, total motility, forward motility and quality of motility were higher from 1 to 6 h post-injection (Fig. 1). The pairwise comparison within the 10 IU hCG treatment indicated that total motility was higher (*p* ≤ 0.027) at 2, 3 and 4 h compared to 6 h (84% vs. 70%). In comparison, the 15 IU hCG treatment maintained a higher total motility (*p* ≤ 0.002) up to the 6 h time point and subsequently decreased in later collections (82% vs 70%). Forward motility followed the same trend in that the 10 IU hCG treatment decreasing after 3 h (53% at 3 h vs. 22% at 6 h), although no statistical differences were found. A peak in forward motility was found at 6 h for the 15 IU hCG treatment (61%) and was higher (*p* ≤ 0.015) than other time points. Similarly, in the 10 IU hCG treatment, quality of motility was highest (*p* ≤ 0.03) at 3 h and decreased by the 6 h collection (2.4 vs. 1.0) whereas in the 15 IU hCG treatment, quality of motility decreased (*p* ≤ 0.018) after 6 h. Sperm obtained at 24 h after hormone injection had slightly lower total motility, forward motility and quality of motility in all hormone treatments but differences were not found to sperm parameters obtained at 7, 8 and 9 h post-treatment. Concentration of spermatozoa showed no differences across time (7.3 × 10^6^ ± 0.5 spermatozoa/mL) although a drop occurred at 24 h post-hormone treatment for both the 10 and 15 IU hCG treatments, when compared with the 6–7 h time point (*p* ≤ 0.024).

### Experiment 2: Effect of cold storage on sperm parameters over time

**Fig. 2:**
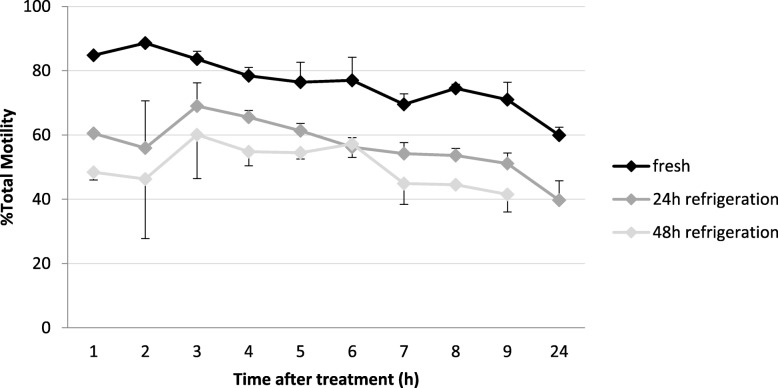
Percentage of total sperm motility in fresh samples (black line) and stored at 5 °C over 24 (drak grey) and 48 h (pale grey). Values are means ± SEM. *N* = 10

The models showed that cold storage at 5 **°**C, time post-hormone treatment and the interaction between the two were significant predictors (*p* < 0.001) for total motility and forward motility, whereas hormone concentration and the interaction between concentration and cold storage were not. Fresh spermic urine samples collected at all time points presented higher total motility (*p* ≤ 0.042) compared to those stored for 24 (78% vs. 58%) and 48 h (78% vs. 50%) at 5 **°**C (Fig. 2). Moreover, total motility was higher at 24 h than at 48 h of refrigeration in several time points. In comparison, forward motility was less affected by storage at 5 **°**C and decreased by 10% after 24 h of cold storage and another 10% decrease by 48 h of storage. Therefore, forward motility from freshly collected sperm did not differ to samples kept for 24 h at 5 °C (40% vs. 32%) but forward motility was higher (*p* ≤ 0.039) in fresh samples than in 48 h refrigerated samples (40% vs. 23%) at almost all time points following hormone treatment. When quality of motility was analysed, the model indicated that hormone concentration, cold storage, time post-hormone treatment and the interaction between cold storage and time post-hormone treatment were significant, but not the interaction between concentration and cold storage. Correspondingly, samples obtained during the first time points were more severely affected by cold storage than samples obtained later. Quality of motility decreased (*p* ≤ 0.027) after 24 h of cold storage in samples obtained during the first 4 h for the 10 IU hCG treatment. However, samples obtained after 5 h post-hormone treatment, showed that quality of motility of freshly collected sperm samples was low and not significantly affected by cold storage. In the 15 IU hCG hormone treatment, quality of motility decreased (*p* ≤ 0.037) after 24 h of cold storage in all samples, except for those obtained at 3 and 7 h post-hormone treatment.

### Experiment 3: Frequency of hCG treatment on sperm parameters

**Fig. 3:**
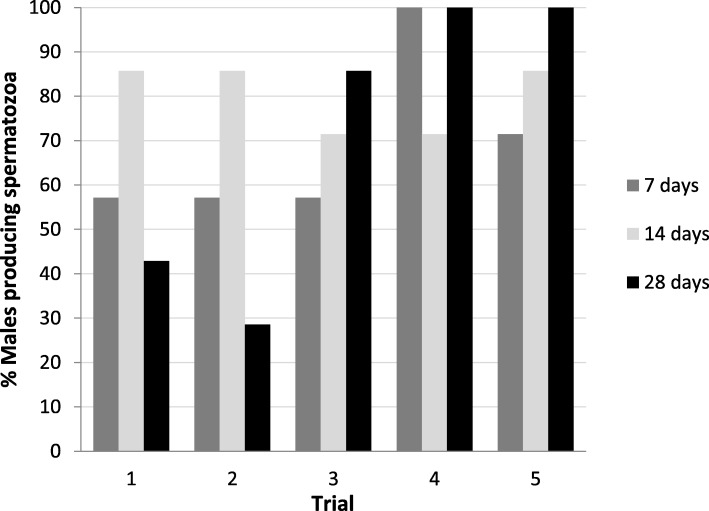
Percentage of males presenting spermatozoa in urine after administration of 10 IU hCG/g BW subjected to three frequencies of hormonal treatment (7, 14, and 28 days). *N* = 7

There were no differences (*p* > 0.05) between weights and SVL of male toads between initiation of the trials and completion of the hormone frequency trials, regardless of treatment group (Table 1). Some males had aspermic urine in some time points or trials, however, all males showed sperm production in at least two trials. None of the males in the biweekly and monthly hormone administration treatments had sperm before the injection in any trial. In contrast, two males in the weekly hormone frequency presented spermatozoa before the hormone treatment in the last trial (week 5). No differences (*p* > 0.05) were found in the percentage of responding males (presenting spermatozoa) among frequencies (Fig. 3). For the monthly frequency treatment group, trial 2 had a lower (*p* = 0.003) percentage of responding males than trials 4 and 5. No differences (p > 0.05) were found between frequencies for sperm parameters in trial 1 indicating similar sperm quality among groups at the beginning of the experiment.

**Fig. 4:**
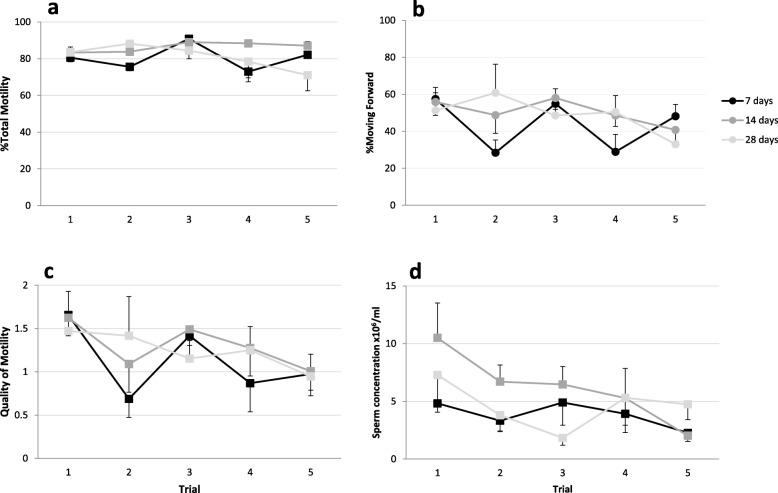
Sperm parameters from male toads treated with 10 IU hCG /g BW across 5 trials and exposed to three different frequencies of hormone administration (7, 14, and 28 days); **a** Percentage of total motility, **b** percentage of sperm moving forward, **c** quality of motility; and **d** sperm concentration. Values are means ± SEM. N = 7

Frequency, trial number and the interaction of both affected the percentage of total motility (*p* ≤ 0.019). The weekly frequency treatment group showed a lower (*p* = 0.001) total motility than the biweekly treatment group. When trials were compared within frequency, no differences in total motility were found within the biweekly and monthly treatment groups. The weekly frequency group had two decreases (*p* < 0.008) in total motility in trials 2 and 4 (Fig. 4). Forward motility was affected by the interaction between frequency and trial (*p* < 0.001). The higher the frequency of treatment the higher the variation on forward motility between consecutives trails. When the analysis was performed separately inside each frequency, trial did not affect (*p* < 0.05) forward motility in the biweekly and monthly frequency groups, whereas in the weekly hormone treatment group forward motility was affected (*p* < 0.003) by trial. Hormone trials and the interaction between trial and frequency was found to impact the quality of motility and sperm concentration (*p* ≤ 0.011). Spermic urine was found to have a lower (*p* = 0.011) quality of motility and sperm concentration in the last trial compared to the first one. Quality of motility was affected by trial in the three frequencies and, similar to forward motility, the higher the frequency the higher the differences in quality of motility. Sperm concentration was higher (*p* ≤ 0.041) in the first trial than in trials 3 and 5 but no differences in spermatozoa concentration where found among other trials.

## Discussion

Although ART development and implementation for amphibian captive breeding programs is a standard practice for some United States and Australian amphibians, very little has been developed for bufonids in other parts of the world. As a consequence, we know less about the reproductive biology of European and Asian amphibian species. Here, we report on the first development of a protocol to stimulate spermiation using exogenous hormones in male natterjack toads as a model for other endangered Eurasian bufonids.

The three concentrations of hCG (5, 10 and 15 IU/g BW) we tested were selected from previous published results on other bufonids [16, 22, 23]. Some studies for sperm collection in anurans used a standardised concentration of hormone regardless of individual body weight [15, 16, 20, 22, 41], whereas other investigators based hormone concentration strictly on a body weight basis [18, 25, 42]. The concentration of hCG we used in our study (5–15 IU/g BW) is similar in range to these previous investigations. The three concentrations we chose to test all produced spermatozoa in *E. calamita* with similar quality; although, our results indicated that 10 and 15 IU were more effective in stimulating spermiation than the lower concentration. We found that males treated with 10 IU in experiment 1 resulted in 100% of males presenting sperm, whereas in experiment 3 less than 70% of toads had spermatozoa in trail 1. This suggest that other factors may be modulating the sensitivity to exogenous hormone treatment. For examples, the natural hormonal status of animals before treatment could affect the sensitivity to hormone stimulation, although the relationship between endogenous hormone levels and the additive response of exogenous hormone stimulation have not been examined. It is possible that seasonality and/or captivity factors (e.g. environmental conditions or nutrition) could be modulating the response to exogenous hormone stimulation. Also, the spermiation response to hCG injections seems to varied among different species of bufonids. In *A. boreas*, researchers found that 300 IU (representing an average of 6.77 IU hCG/g BW) stimulated spermiation in 100% of males [16]. However, in *A. baxteri*, the administration of a similar hCG dose (approximately 7.6 IU/g BW) induced spermiation in about 80% of the males [13] and in *R. marina* treatment with 1000 IU hCG (between 7 and 13 IU/g BW) produced sperm in 75% of toads [22].

Although no effect of hCG concentration was observed on sperm quality, the variation in sperm parameters over time within each hormone treatment was different. Sperm obtained after treatment with 10 IU hCG showed best sperm quantity and quality from 1 to 4 h after hormone treatment while spermic urine obtained following treatment with 15 IU hCG had better sperm quality up to 6 h after treatment. Sperm concentration was nearly constant for the first 9 h regardless of treatment. In comparison, *A. baxteri* and *A. americanus* motility was similar from 5 to 13 h post-injection with 5 IU hCG/g BW and concentration peaked between 7 to 9 h [13, 16]. For *A. fowleri* sperm concentration peaked earlier at 5 h post-hormone treatment [4]. In contrast, *R. marina* showed no differences in motility and concentration at 3, 6 and 12 h post-hormone treatment with 7 to 13 IU/g BW hCG [22]. From our results, we found that using 10 IU hCG/g BW for stimulation of spermiation in *E. calamita* worked the best when sperm is going to be obtained outside the breeding season and during the first 4 hours after injection. If sperm collections will be stretched out over a longer period of time, we would likely select the15 IU hCG concentration given its prolonged effect.

Injection of gonadotropins produce swelling of Sertoli cells by an increase in water content. Over time, the cell swelling decreases and eliminates the apical invaginations that retained the sperm with a concurrent release of spermatozoa and fluid into the lumen of seminiferous tubules [19, 43]. The sperm-release effect of gonadotropins on sertoli cells last for many hours; for *E. calamita* sperm release lasted at least 9 h as has been shown for other bufonids, and probably decreased slowly over 20–30 h. The sperm release following hormone treatment easily covers the natural time period *E. calamita* would be in amplexus. We are uncertain how sperm quality we collected through hormone treatment would compare to sperm collected from a naturally amplexed male. To our knowledge, such comparisons have yet to be done. However, time points with best sperm quality found in this study was in accordance with timing for natural reproduction in the natterjack toads as amplexus and spawning typically last for 3 to 5 h in this species [44].

*E. calamita* sperm cold stored at 5 °C was motile up to 48 h, although the largest drop in total motility occurred during the first 24 h (15–20%), compared to the second half of storage. These results are similar to other studies on short-term cold storage of anuran spermatozoa. Cold-stored spermic urine collected after hormonal treatment in an American bufonid, *A. fowleri,* showed a similar drop of 25% in total motility after 24 h at 5 °C [15] and refrigerated spermic urine in that species retained fertilizing capability for more than 8 days (Germano et al., unpublished results). Aeration of spermic urine before storage at 5 °C vs. non-aeration resulted in a lower drop in motility in *A. fowleri* [15] and in a non-bufonid toad [45]. Although the effect on fertility is unknown it is likely that aeration or oxygenation could improve motility maintenance also in *E. calamita* cold stored spermatozoa if administered directly before storage.

We found that sperm characteristics were affected by the frequency of the hormonal treatment. With a higher frequency of hormone stimulation (e.g. weekly), we observed a lower percentage of total motility and forward motility. Moreover, weekly frequency presented a higher variability in sperm motility between consecutive trials. In contrast, animals in the biweekly and monthly treatment group showed no effect on sperm parameters. Therefore, consecutive hCG hormone treatments should be spaced for at least 2 weeks apart to maintain better motility. Sperm concentration decreased after the first trial in all frequencies. Similarly, a decrease in sperm concentration was observed using a higher frequency of hormone stimulation when hCG was administered twice a week in *A. fowleri* [33], or LHRH given twice a week to *R. marina* [34] and in *Rana pipiens* treated for 8 consecutive days with hCG [46]. One hypothesis for the lower sperm count from more frequent hormone stimulations is that a down regulation of hormone receptors occurred such that hormone responsiveness is attenuated [33]. This explanation is also partially supported by our data. Interestingly, during the last trial of the weekly frequency treatment group, two males presented sperm in their urine before the hormone injection. Furthermore, after the hormone treatment no spermatozoa were found in the urine of one of the males. Similarly, injection of hCG in wild *Litoria ewingii* that presented sperm before hormone treatment showed that sperm was no longer present in the urine following hormone administration (J. Germano, personal communication). In both cases, the presence of sperm pre-treatment is likely due to a high level of endogenous gonadotropins and the additional exogenous hormone administration probably produced a negative feedback, which inhibited spermiation. However, other explanations such as sperm depletion or suppression of spermatogenesis that may be due to lower testosterone levels could also be a factor [33]. More than 60 years ago, it was suggested that a repose of at least 10 days between consecutive hormonal treatments are required in *Bufo bufo* during the summer and that this rest period could be longer in the winter [47]. We found a similar effect of frequency of treatment on sperm production outside of the normal breeding season and further studies are warranted.

Taking these results together, the dynamics of sperm quality and quantity after hormonal induction of spermiation in *E. calamita* is similar to related species but species specific as proposed previously [4, 18] thus, it could be suggested that similar protocols could be develop in other Eurasian or African bufonids.

## Conclusion

This is the first time that a protocol for stimulation of spermiation has been developed in a non-American bufonid. We found that at specific concentrations, exogenous hCG administration outside the breeding season successfully induced spermiation within a couple of hours in 85% of the males. Moreover, sperm quality was affected by the interaction of hormone concentration and time post-treatment and showed a steady peak in sperm production between 2 to 6 h. Sperm concentration declined by 24 h post-hormone administration. Importantly, we found that natterjack toad sperm could be cold stored up to 48 h post-hormone administration and still show reasonable motility for potential fertilizations. Also, sperm quality was negatively affected by increasing the frequency of hormone administration. Therefore, evaluating the effect of hormone concentration, time and frequency of hormonal treatment on sperm parameters, relative to quantity and quality of sperm, should be considered when developing a hormone stimulation protocol for at-risk Eurasian amphibian species that are in need of ART.

## Data Availability

The datasets used and/or analysed during the current study are available from the corresponding author on reasonable request.

## Abbreviations

ART: Assisted reproductive technologies
BW: Body weight
GnRH: Gonadotropin releasing hormone
hCG: Human Chorionic Gonadotropin
LHRH: Luteininzing hormone-releasing hormone
